# Seedbank persistence and emergence pattern of *Argemone mexicana*, *Rapistrum rugosum* and *Sonchus oleraceus* in the eastern grain region of Australia

**DOI:** 10.1038/s41598-021-97614-8

**Published:** 2021-09-10

**Authors:** Sudheesh Manalil, Bhagirath Singh Chauhan

**Affiliations:** 1 Queensland Alliance for Agriculture and Food Innovation (QAAFI), The University of Queensland, Gatton, QLD 4343 Australia; 2 School of Agriculture and Environment, The University of Western Australia, Perth, Australia; 3 Amrita Vishwa Vidyapeetham, Coimbatore, India; 4 School of Agriculture and Food Sciences (SAFS), The University of Queensland, Gatton, QLD 4343 Australia; 5 Adjunct Faculty, Chaudhary Charan Singh Haryana Agricultural University (CCSHAU), Hisar, Haryana 125004 India

**Keywords:** Plant sciences, Environmental sciences

## Abstract

A thorough understanding of the emergence pattern and persistence of weed seeds is a prerequisite in framing appropriate weed management options for noxious weeds. In a study conducted at the University of Queensland, Australia, the emergence and seed persistence behavior of three major weeds *Sonchus oleraceous, Rapistrum rugosum,* and *Argemone mexicana* were explored with seeds collected from Gatton and St George, Queensland, Australia, with an average annual rainfall of 760 and 470 mm, respectively. Seed persistence was evaluated by placing seeds at the surface layer (0 cm) or buried at 2 and 10 cm depths enclosed in nylon mesh bags and examined their viability for 42 months. In another study, the emergence pattern of four populations, each from these two locations, was evaluated under a rainfed environment in trays. In the mesh-bag study, rapid depletion of seed viability of *S. oleraceous* from the surface layer (within 18 months) and lack of seed persistence beyond two years from 2 and 10 cm depths were observed. In trays, *S. oleraceous* germinated 3 months after seeding in response to summer rains and there was progressive germination throughout the winter season reaching cumulative germination ranging from 22 to 29% for all the populations. In the mesh-bag study, it took about 30 months for the viability of seeds of *R. rugosum* to deplete at the surface layer and a proportion of seeds (5 to 13%) remained viable at 2 and 10 cm depths even at 42 months. Although fresh seeds of *R. rugosum* exhibit dormancy imposed due to the hard seed coat, a proportion of seeds germinated during the summer months in response to summer rains. Rapid loss of seed viability was observed for *A. mexicana* from the surface layer; however, more than 30% of the seeds were persistent at 2 and 10 cm depths at 42 months. Notably, poor emergence was observed for *A. mexicana* in trays and that was mostly confined to the winter season.

## Introduction

Annual sowthistle (*Sonchus oleraceus* L.), turnip weed [*Rapistrum rugosum* (L.) All.] and Mexican poppy (*Argemone mexicana* L.) are three major winter weeds of agricultural landscapes across the world^1–8^. These weeds are quite predominant under the conservation agricultural systems of Australia and can invade agricultural landscapes and environments rapidly due to their superior competitiveness, high seed production ability, and their biological features^2,9,10^. *Rapistrum rugosum* and *A. mexicana* are generally confined to winter growing conditions, exhibiting a high level of competitiveness and reproductive potential, though they can emerge and set some seeds during the post-winter season^2,7,10–12^. Though *S. oleraceus* is also a predominant weed of winter seasons, unlike the other two weeds, it can emerge and grow well throughout the year^2,13,14^*.* These three weeds can cause a substantial yield reduction in crops and can be a perennial problem as growers find it difficult to manage these weeds once they have infested the crop^7–9,15,16^.

Germination ecology studies of *S. oleraceous* (Asteraceae family) indicated that this weed could germinate under a wide range of pH, salinity and temperature conditions, and seeds could germinate immediately after maturity as they lack dormancy^2,6,16,17^. Also, *S. oleraceous* could produce a substantial number of seeds and can be dispersed through wind prior to crop harvest making management quite difficult^15^. The biological and reproductive potential of this weed makes it a year-round problem^13,15–17^. If unattended, the post fallow phase following a winter crop can be a breeding ground for this weed as it can flourish under fallows utilizing the residual fertility and soil moisture^2,15,18^. A density of about 50 plants m^-2^ resulted in a yield reduction of 50% in wheat^15^. Moreover, many herbicide-resistant populations were observed in the cotton and grain cropping regions of Australia^13,14^.

*Rapistrum rugosum* is an annual broadleaf weed from the Brassicaceae family and is a major agricultural and environmental weed in countries including Australia, the USA, Russia, and Iran^9,19–21^. A high level of competitiveness, abundant seed production, and dormancy induced by the seed coat can attribute to the invasive potential of this weed^1,9,11^. *R. rugosum* is a highly competitive weed; about 20 plants m^-2^ caused a yield reduction of 50% in wheat^9^. This weed is generally confined to the winter season. However, when emerged in the latter part of the winter season, plants were short in stature and produced fewer seeds indicating a photoperiodic response in this weed^2,12^. Seed retention on the plant at harvest contributes to the potential for weed seed destruction during the harvest time^9^. This weed can develop herbicide resistance^3,22^ and lack of integrated management without a multipronged approach can enhance *R. rugosum* infestation in the coming years^1,9,21^.

*Argemone mexicana* is an annual broadleaf weed from the Papaveraceae family. It is a global agricultural weed that can be both an agricultural and environmental problem, can lead to yield reductions in crops, and can be poisonous to human beings and cattle^7,8,10^. *A. mexicana* is quite prevalent in the cotton tracts and grain cropping regions of Australia^13,23^. Once infested, the infestation can last for many years. Besides causing crop yield reductions, weed management and cultural operations can be difficult due to its spiny nature (CottonInfo 2014; Manalil et al*.* 2017). Although poor competitiveness of this weed in wheat is observed^9^, this weed can be a problem in the chickpea growing tracts and fallow regions^24^. Knowledge gaps exist on the seed biology of this weed especially on the fate and germination pattern under field conditions.

Although studies on seed ecology were performed in the region especially on the emergence potential of these weeds under different environments^1,11^, the persistence of these weeds in the field conditions and their emergence pattern is not fully understood and explored through scientific studies. The dormancy pattern and persistence can vary under field conditions and such information is highly important in framing ideal weed management options. To bridge the knowledge gaps in the emergence pattern of these invasive weeds, a study was conducted to explore the seed persistence and emergence pattern of these weeds under field conditions.

## Materials and methods

### Seed collection

**Figure 1:**
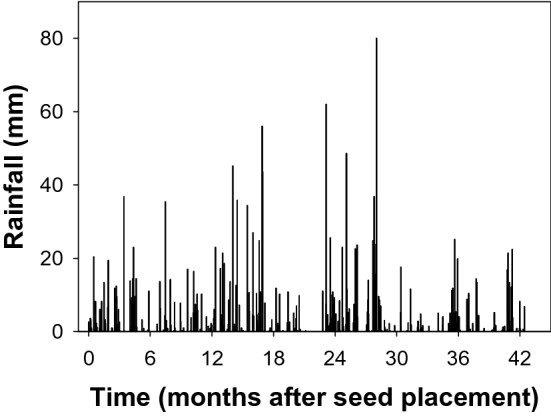
Rainfall at Gatton corresponds to seed burial in nylon mesh bags starting from November 2015 to April 2019.

This study complies with relevant institutional, national, and international guidelines and legislation for using plant material. The study was conducted at the Gatton Research Farm of the University of Queensland, Australia (S 27.538281, E 152.334269). The experiment was established with weed populations of *S. oleraceus, R. rugosum*, and *A. mexicana* collected from the St George and Gatton regions of Queensland, Australia. Four populations of each species were collected in each region including crop fields and adjacent non-cropping areas (Table 1). The authors confirm that the owner of the land gave permission to collect the weed seeds, as well as that the field studies did not involve endangered or protected species. Gatton and St George receive an annual average rainfall of 760 mm and 470 mm, respectively. The locations are characterized by summer dominant rainfall; Gatton and St George receive a share of 40% and 38% of the annual rainfall during summer, respectively. The Gatton trial location received an annual rainfall of 681, 562, 797, 518, and 230 mm in the years 2015, 2016, 2017, 2018, and 2019, respectively (Fig. 1). Although 2019 was a drought year (with 230 mm), 145 mm was received during the study period (study finished in April 2019).

**Table 1:** Geographic locations and cropping history of weed seed collection.

Weed	Region	Population	Situation	GPS coordinates
*Argemone mexicana*	Gatton	AMG1	Chickpea	S27 03.994, E151 04.696
		AMG2	Fenceline	S27 04.016, E151 04.633
		AMG3	Chickpea	S27 32.846, E152 21.232
		AMG4	Fenceline	S27 32.830, E152 21.205
	St George	AMS1	Chickpea	S28 19.326, E148 31.068
		AMS2	Fenceline	S28 19.062, E148 31.152
		AMS3	Wheat	S28 01.222, E148 41.418
		AMS4	Fenceline	S28 01.179, E148 41.766
*Rapistrum rugosum*	Gatton	RRG1	Wheat	S27 33.552, E152 19.443
		RRG2	Fenceline	S27 33.554, E152 19.409
		RRG3	Wheat	S27 49.097, E151 30.699
		RRG4	Fenceline	S27 49.094, E151 30.686
	St George	RRS1	Chickpea	S28 11.104, E148 38.054
		RRS2	Fenceline	S28 11.095, E148 38.056
		RRS3	Wheat	S28 14.495, E148 39.633
		RRS4	Fenceline	S28 14.481, E148 39.711
*Sonchus oleraceus*	Gatton	SOG1	Wheat	S27 33.551, E152 19.412
		SOG2	Fenceline	S27 33.554, E152 19.409
		SOG3	Wheat	S27 49.097, E151 30.699
		SOG4	Fenceline	S27 33.185, E152 20.112
	St George	SOS1	Fenceline	S28 14.474, E148 39.604
		SOS2	Chickpea	S28 14.489, E148 39.629
		SOS3	Chickpea	S28 11.089, E148 38.059
		SOS4	Fenceline	S28 11.118, E148 38.041

Mature weed seeds/fruits were collected by shaking the inflorescences into a tray, placed into a paper bag, and stapled. A handheld GPS was used to record the coordinates of the collection site. On arrival, the paper bags were kept under a ventilated rainout shelter facility at Gatton and cleaned seeds were kept in paper bags and stored in the rainout shelter facility.

### Seed persistence study using mesh-bags under field conditions

Fifty seeds of *S. oleraceus, R. rugosum,* and *A. mexicana* from St. George and Gatton (two populations) were placed in mesh bags (nylon bags) and buried at 0, 2, and 10 cm depths at Gatton (November 2015). Before placing in mesh bags, seeds were cleaned and winnowed through a custom-made seed vacuum cleaner and examined through a seed X-Ray unit (Faxitron seed X-ray unit) to ensure that seeds were of high quality and filled. Bags were exhumed at different times (0, 3, 6, 12, 18, 24, 30, 36, and 42 months after burial) and examined in the laboratory. Once exhumed, germination of seeds was assessed by placing all the recovered seeds in a 9 cm diameter Petri dish with two Whatman No.1 filter papers and moistened with 5 ml of distilled water. Petri dishes were covered with zip-lock plastic bags to minimize moisture loss and placed in an incubator set at day/night alternating temperatures of 20/10 °C for three weeks. Germination was recorded, the ungerminated seeds were gently squeezed and the decayed seeds were subtracted from the recovered seeds to calculate the percentage of viable seeds.

### Emergence pattern of weeds in trays under field conditions

**Figure 2:**
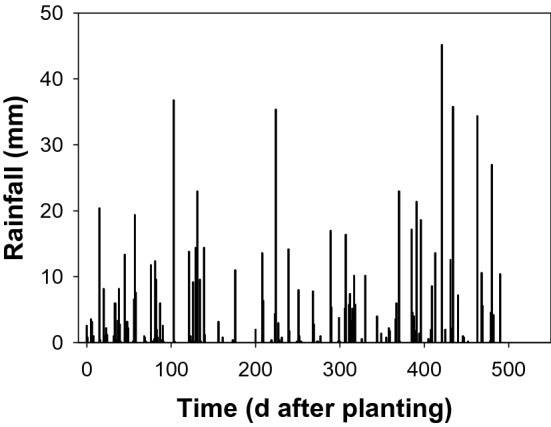
Rainfall at Gatton corresponds to emergence of weeds in trays under a rainfed environment starting from November 2015 to April 2017.

One hundred seeds were spread on the surface of seeding trays filled with a potting mix and placed on the soil surface under field conditions at the Gatton farm of the University of Queensland. Unlike with the field soil, the potting mix was free of weed seeds and therefore, the potting mix was used in trays. Four weed populations each from St George and Gatton were evaluated from November 2015 to May 2017 under the rainfed environment (Fig. 2). There were three replicate trays for each weed population. The emergence of weeds was recorded at a biweekly interval, emerged seedlings were removed from the seed trays, and soil was disturbed to stimulate the germination and emergence of buried weed seeds.

### Statistical analysis

Both studies (seedbank persistence and emergence pattern) were conducted using a randomized complete block design with three replications of each treatment. Analysis of variance (ANOVA) showed that the differences between the populations and the interaction between population and treatment were significant (Genstat 19th Edition); therefore, the data are presented separately for each population. Percentage seed persistence and seed germination was expressed as the mean and the standard error was computed. Data were presented graphically with error bars representing the standard error of means. Graphic representation of the data was done using the SigmaPlot software.

## Results

### Seed persistence and emergence pattern of Sonchus oleraceous

**Figure 3:**
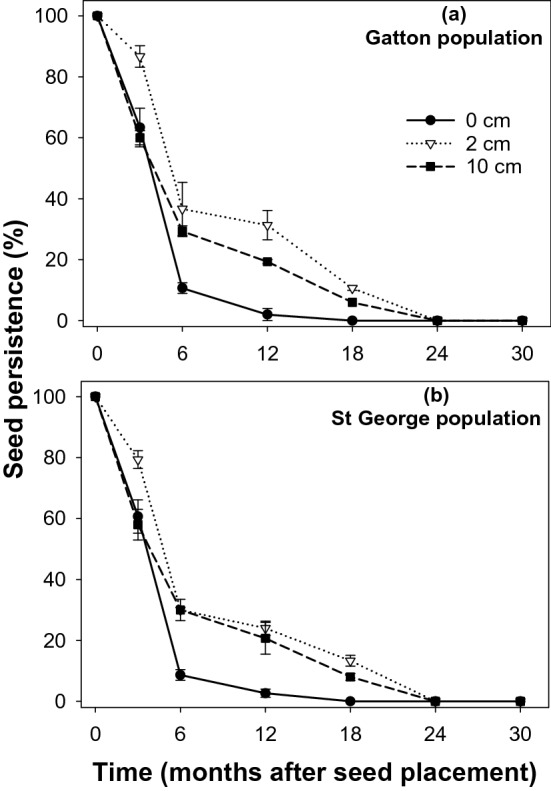
Seed persistence of *Sonchus oleraceous* from Gatton (**a**) and St George (**b**) locations placed in mesh-bags at 0, 2, and 10 cm depths, symbols correspond to mean and error bars are the standard error of means (n = 3).

In the mesh-bag study, at the surface layer (0 cm), 63 and 61% of seeds of *S. oleraceous* were viable for the Gatton and St George populations, respectively, at 3 months (Fig. 3). A quick depletion of *S. oleraceous* seeds was noticed after 3 months, with 2 and 3% viable seeds present at 12 months for the Gatton and St George populations, respectively, and no viable seeds observed at 18 months and afterward. At 2 cm depth, 87 and 79% viable seeds were recovered at 3 months for the Gatton and St George populations, respectively, and at 12 months, 31 and 24% seeds were recovered for the Gatton and St George populations, respectively. All the seeds of *S. oleraceous* were depleted by 24 months. At 10 cm depth, 60 and 58% of seeds were viable at 3 months for the Gatton and St George populations, respectively. At 12 months, the seed viability was dropped to 19 and 21%, and no viable seeds were observed at 24 months and afterward.

**Figure 4:**
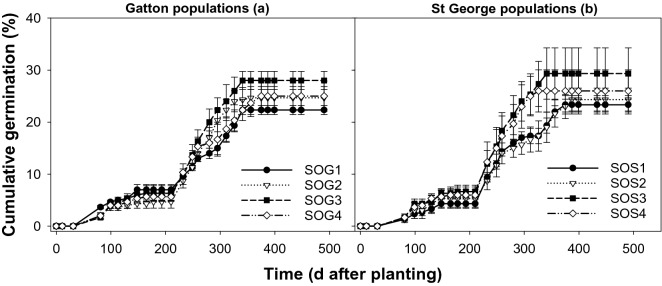
Emergence pattern of *Sonchus oleraceous* populations from Gatton (SOG1-SOG4; a) and St George (SOS1-SOS4; b) in trays under a rainfed environment (November 2015 to April 2017), symbols correspond to mean and error bars are the standard error of means (n = 3).

In trays, the first flush of *S. oleraceous* was observed 81 days after sowing (DAS) in February 2016 coinciding with the major rain event of January and February (Fig. 4). Subsequently, germination progressed over time and the maximum proportion of cumulative germination was observed between 232 (June) to 340 DAS (October) coinciding with the winter growing season. The cumulative germination from all the populations of *S. oleraceous* varied between 22 to 29%.

### Seed persistence and emergence pattern of Rapistrum rugosum

**Figure 5:**
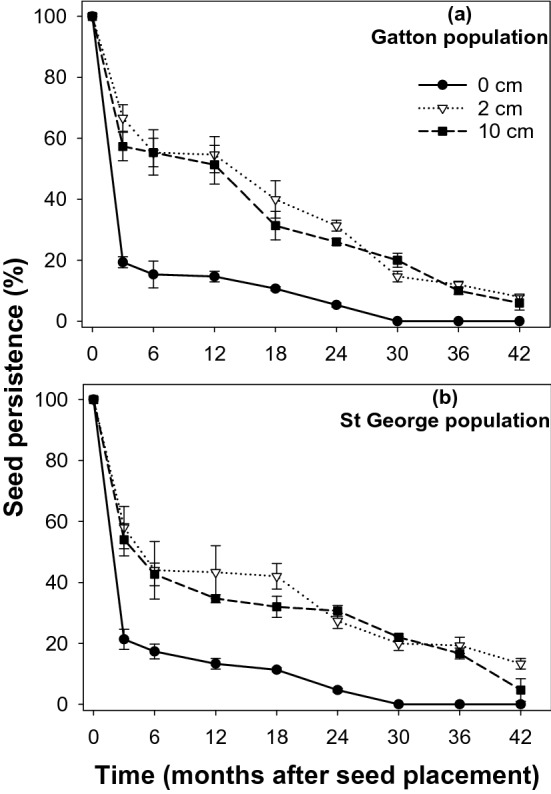
Seed persistence of *Rapistrum rugosum* from Gatton (**a**) and St George (**b**) locations placed in mesh-bags at 0, 2, and 10 cm depths, symbols correspond to mean and error bars are the standard error of means (n = 3).

In the mesh-bag study, at 0 cm depth, 19 to 21% viable seeds of *R. rugosum* were observed after 3 months and there were progressive reductions over time (Fig. 5). At 12 months, 15 and 13% of seeds were viable for the Gatton and St George populations, respectively. At 24 months, 5% of seeds were viable for both the populations for the surface layer, and seeds completely depleted by 30 months. At 2 cm depth, 67 and 58% of seeds were viable at 3 months for the Gatton and St George populations, respectively. At 12 months, 55 and 43% of seeds were viable at Gatton and St George, respectively, and at 24 months, 31 and 27% of seeds were viable for Gatton and St George, respectively. Substantial reductions in the recovered viable seeds were observed at 42 months as there were 8 and 13% of viable seeds at Gatton and St George, respectively. At 10 cm depth, at 3 months, 57 and 54% of seeds were viable for Gatton and St George, respectively. At 12 months, 51 and 35% of seeds were viable for Gatton and St George, respectively, and at 24 months, 26 and 31% of seeds were viable for Gatton and St George, respectively. Substantial reduction in the recovered viable seeds observed at 42 months as observed at 2 cm depth, there were 6 and 5% of viable seeds at Gatton and St George, respectively.

**Figure 6:**
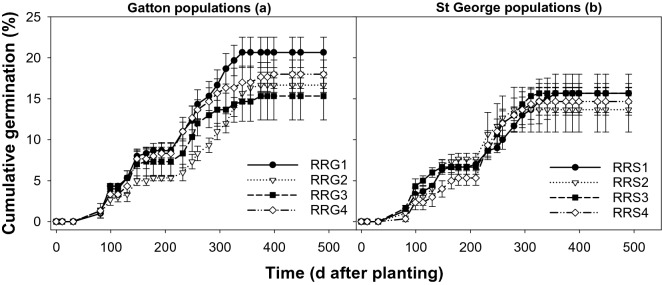
Emergence pattern of *Rapistrum rugosum* from Gatton (RRG1-RRG4; a) and St George (RRS1-RRS4; b) in trays under a rainfed environment (November 2015 to April 2017), symbols correspond to mean and error bars are the standard error of means (n = 3).

As for *S. oleraceous*, the major first flush of *R. rugosum* in trays was observed at 81 DAS in February 2016, coinciding with the major rain event of January and February (Fig. 6). The second flush was observed between 232 to 340 DAS coinciding with the winter growing season. The germination was stabilized by the end of the crop growing season and no germination was observed afterward. The cumulative germination of all the populations varied between 14 and 21%.

### Seed persistence and emergence pattern of Argemone mexicana

**Figure 7:**
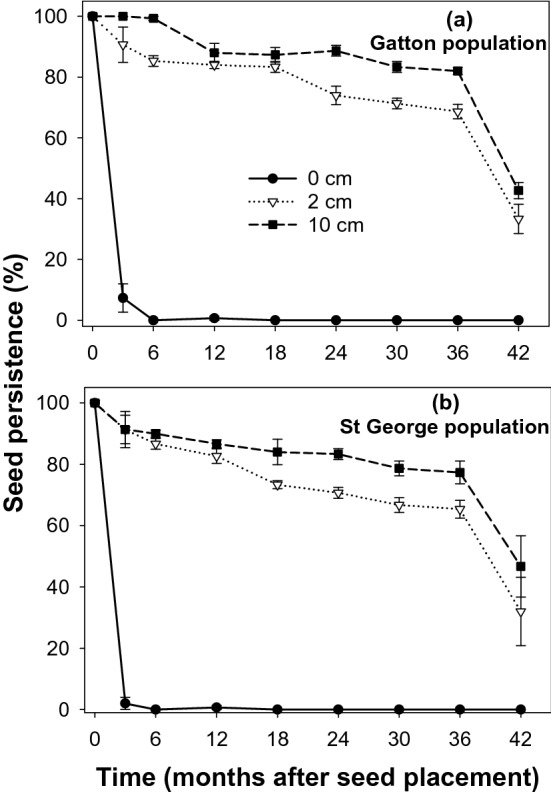
Seed persistence of *Argemone mexicana* seeds from Gatton (**a**) and St George (**b**) locations placed in mesh-bags at 0, 2 and 10 cm depths, symbols correspond to mean and error bars are the standard error of means (n = 3).

In the mesh-bag study, at the surface layer, only 7 and 2% of viable seeds were observed at 3 months after seed placement for Gatton and St George, respectively (Fig. 7). Seeds from the surface layer were split open or disintegrated when gently squeezed with a pair of forceps. At 12 months, less than 1% of viable seeds were viable and seeds fully disintegrated at the surface layer by 18 months. At 2 cm depth, > 90% of seeds were observed at 3 months for both populations, and 84 and 83% of seeds were viable at 12 months for the Gatton and St George populations, respectively. At 24 months, 74 and 71% of seeds were recovered at 2 cm for Gatton and St George, respectively. At 42 months, 32–33% of seeds were viable for both populations. For both populations at 10 cm, 100% of seeds were viable at 3 months and 87–88% of seeds were viable at 12 months. At 24 months, 88 and 83% of seeds were viable for Gatton and St George, respectively, and 43 and 47% of seeds were viable at 42 months for the Gatton and St George populations, respectively.

**Figure 8:**
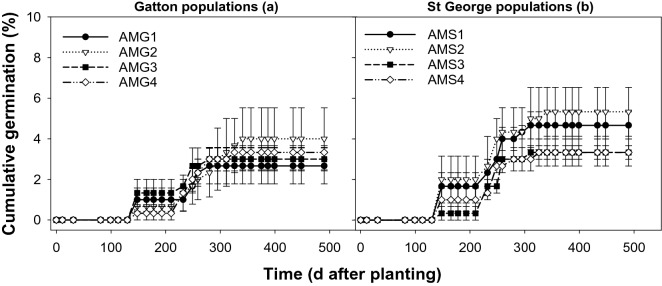
Emergence pattern of *Argemone mexicana* populations from Gatton (AMG1-AMG4; a) and St George (AMS1-AMS4; b) in trays in trays under a rainfed environment (November 2015 to April 2017), symbols correspond to mean and error bars are the standard error of means (n = 3).

The first emergence flush of *A. mexicana* was noticed 148 DAS in March 2016 (Fig. 8). Although there was good summer rainfall of 93 and 80 mm in January and February of 2016, respectively, no germination was observed during these months, indicating freshly harvested seeds were dormant for around 5 months in the field conditions. Observed emergence at 148 DAS was less than 2% and the next flush was observed 248 DAS corresponding to July (winter season), and the cumulative emergence of all the populations was less than 6%. No further emergence was noticed until the completion of the experiment.

## Discussion

Rapid depletion in seed viability of *S. oleraceous* from the surface layer and lack of persistence beyond 2 years at 2 and 10 cm depths provides some insights for the management of an infested paddock. Diversified weed management options to reduce the weed seedbank enrichment, including competitive crops in rotation, narrow row spacing, high seeding rate, soil inversion tillage, herbicide rotation and use of herbicide mixtures may reduce the infestation of this species. Unlike *S. oleraceous* that depleted in the surface soil layer within 18 months, it took around 30 months for *R. rugosum* seeds to lose all viability at the surface layer and a proportion of seeds were still viable even after 42 months at 2 and 10 cm depths. Considering the high competitiveness of *R. rugosum*^9^, utmost care should be taken to manage this weed by integrating both chemical and non-chemical methods with special emphasis to minimize the weed seedbank enrichment. Integration of inversion tillage will not yield desired results due to the slow depletion of seed viability when buried. *R. rugosum* exhibits continuous flowering and seed set but a high level of seed retention offers opportunities for destroying the weed seeds at wheat crop harvest^9,12^.

The emergence of *S. oleraceous* and *R. rugosum* in trays in early February (81 DAS) in response to summer rains indicate a proportion of the seeds could germinate well ahead of the winter season. However, the emergence of *A. mexicana* was later and occurred at 148 DAS in March. *S. oleraceous* is characterized by a year-round germination pattern and freshly harvested seeds were devoid of dormancy, indicating it could germinate when the environment is conducive for germination^2,6,17^. However, in the case of *R. rugosum*, the seed coat imposes dormancy, freshly harvested seeds exhibit 100% dormancy, and once silique is removed seeds germinate^1,11^. The results indicate that a portion of freshly harvested seeds of *R. rugosum* released dormancy under the field environment and emerged along with *S. oleraceous*. In the case of *A. mexicana,* the germination in trays was poor and the observed germination was confined to early winter and winter growing seasons indicating release of dormancy under field conditions will be towards the winter crop growing season.

For *A. mexicana*, rapid depletion of seeds was observed from the surface layer (Fig. 7). The reason for the quick disintegration of seeds placed at the surface layer in mesh-bags is not clear; however, nylon bags restricting the movement of seeds to bottom layers and exposure to sunlight, alternate wetting, and thawing or infestation of insects or diseases could be the possible reasons for the rapid disintegration of seeds. The germination in trays was also poor indicating either seeds were disintegrated as observed in nylon bags or moved to bottom layers and persisted or remained dormant; a high level of seed persistence was observed at 2 and 10 cm depths. A previous study confirms the poor germination of *A. mexicana*, as a majority of seeds did not germinate during their first season after shedding due to strong dormancy and seeds tend to be persistent for several years^8^. High persistence of buried seeds of *A. mexicana* warrants careful management of the weed. Weed seeds could easily move in water crevices, acquire dormancy, and persist for several years (CottonInfo 2014; Karlsson et al. 2003). Due to these reasons, soil inversion tillage may not yield the desired result as it is impossible to bring all the buried seeds to the soil surface and seeds that are covered by soil up to 2 cm depth showed a substantial level of persistence. Competition from wheat leads to the suppression of this weed^9^, indicating the possibility to enhance crop competitiveness as a strategy that could be integrated with other weed management options.
